# Integrated Return-To-Field and Targeted Trap-Neuter-Vaccinate-Return Programs Result in Reductions of Feline Intake and Euthanasia at Six Municipal Animal Shelters

**DOI:** 10.3389/fvets.2019.00077

**Published:** 2019-03-21

**Authors:** Daniel D. Spehar, Peter J. Wolf

**Affiliations:** ^1^Independent Researcher, Cleveland, OH, United States; ^2^Best Friends Animal Society, Kanab, UT, United States

**Keywords:** return-to-field (RTF), trap-neuter-vaccinate-return (TNVR), targeted TNVR, unowned free-roaming cats, community cat program (CCP), feline intake, feline euthanasia, animal sheltering

## Abstract

For decades, animal shelters in the U.S. have sought to reduce the number of cats that are impounded and euthanized. Since the 1990s, low-cost sterilization campaigns aimed at owned cats have achieved varying levels of success in meeting these objectives. Over a similar time period, the use of trap-neuter-vaccinate-return (TNVR), as a humane alternative to the lethal management of stray and feral cats, has proliferated. Because of the limited scope of many TNVR programs, the impacts of such efforts on shelter metrics have often proven difficult to measure. In the past decade, two new variants of TNVR, return-to-field (RTF) and high-impact targeting, have exhibited the capacity to contribute to significant reductions in shelter intake and euthanasia. The present study examines changes in feline intake and euthanasia, as well as impacts on associated metrics, at municipal shelters located in six diverse U.S. communities after integrated programs of RTF and targeted TNVR (collectively termed “community cat programs,” CCPs) were implemented. A total of 72,970 cats were enrolled in six 3-year CCPs, 71,311 of whom (98%) were sterilized, vaccinated, and returned to their location of capture or adopted. A median reduction of 32% in feline intake, as well as a median decline of 83% in feline euthanasia occurred across the six CCPs; median feline live-release rate increased by 53% as a result of these simultaneous declines in cat admissions and euthanasia. The integration of RTF and targeted TNVR protocols appears to result in greater feline intake and euthanasia reductions than programs lacking such an integrated approach.

## Introduction

Unlike some countries (e.g., Italy), the U.S. has no national laws governing the management of free-roaming domestic cats; relevant local and state laws vary considerably. In addition, each animal shelter typically has its own relevant policies and guidelines. The focus of the present study is the impact of relevant policy changes—not the laws—regarding the admission and disposition of community cats following the implementation of innovative programs intended to humanely manage the population of unowned, free-roaming cats (often referred to as “stray” or “feral,” terms typically used interchangeably in the U.S. and Canada, but referred to as “community cats” throughout this paper). The legal aspects of such programs have recently been taken up by others, including the American Bar Association (1, 2).

Open-admission shelters, facilities that generally accept any animal in need, including those with little chance of being rehomed due to issues of age, health, or temperament (3), are often either operated directly by municipalities or by private organizations under government contract. In recent decades, municipalities across the United States have expended substantial resources aimed at reducing the number of cats admitted to and euthanized at such shelters. Government-funded low-cost (or no-cost) sterilization campaigns, often focused on owned cats in underserved communities, have been associated with reductions in feline intake and euthanasia (4–6). Nevertheless, data going back to the 1990s from a number of states have revealed varying trends in these shelter metrics (7–9). A proliferation in the use of trap-neuter-vaccinate-return (TNVR) as a method of managing community cats has occurred over a similar time period. Declines in colony size associated with such programs (10–12), including the elimination of individual colonies (13, 14), and reduction (15) or elimination (16) of kitten births, have been documented. Nevertheless, because TNVR has been historically conducted on a limited scale, often at the colony level, the impact of such programs on the intake and euthanasia of cats at municipal shelters is unclear.

**Figure 1:**
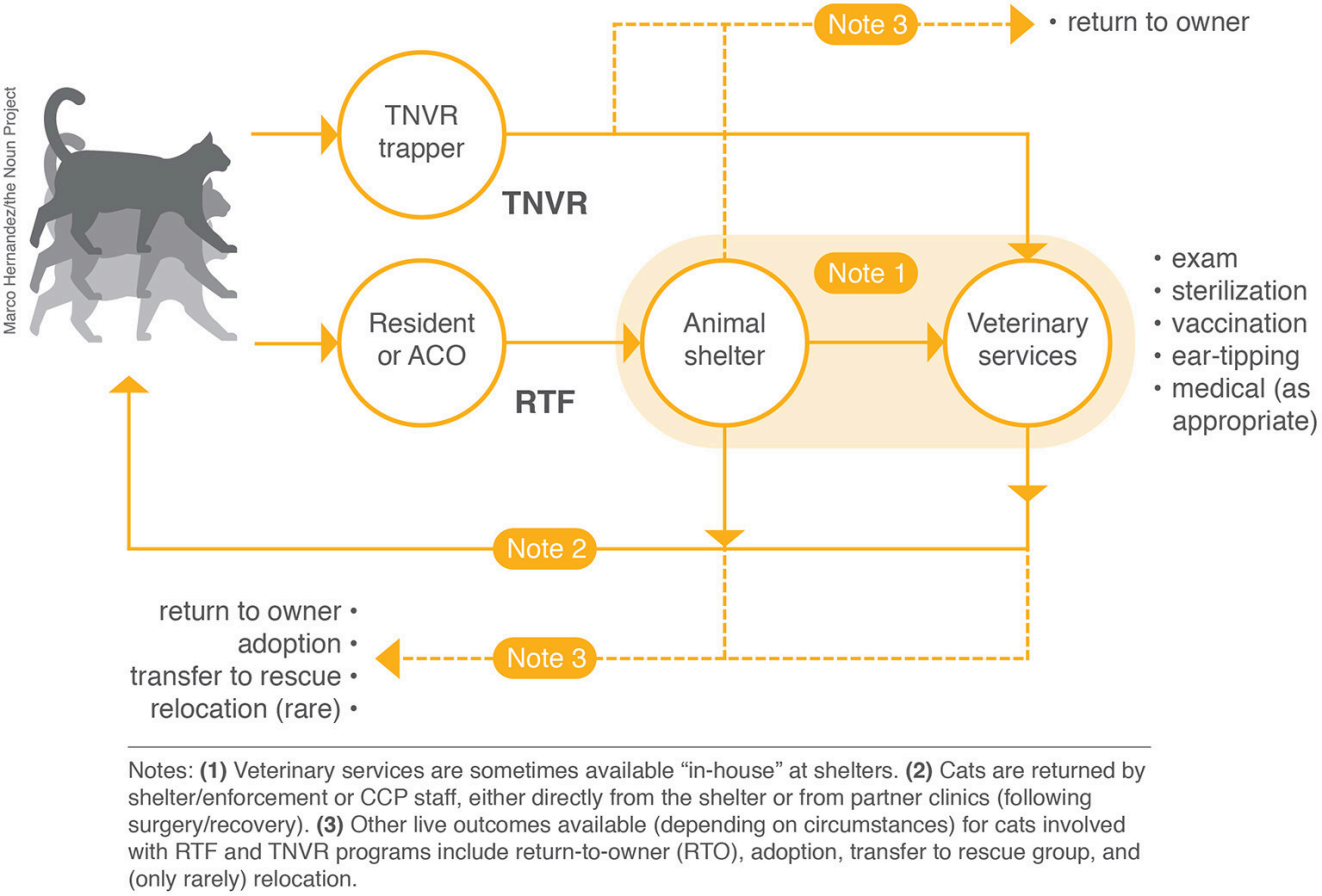
Visual representations of TNVR and RTF programs.

Two new, scaled-up variants of TNVR, high-impact targeting and return-to-field (RTF), have been developed over the past decade and appear to have transformative potential for reducing the intake and euthanasia of cats at municipal shelters. Targeted TNVR is a systematic approach whereby efforts to trap, sterilize, vaccinate, and return cats are concentrated in areas known to have a high-density of community cats; these targeted areas are also often a source of high feline intake at municipal shelters. RTF programs (sometimes called Feral Freedom or shelter-neuter-return, SNR) are similar in that they involve the sterilization, vaccination, and return of cats. However, these programs are shelter-based rather than community-based; RTF programs are essentially TNVR programs for cats designated as “strays” upon admission to the shelter (either brought by residents or impounded by enforcement staff). RTF programs are, like TNVR programs, implemented with the 2-fold aim of reducing (i) the number of cats who, either due to temperament or lack of shelter space, would otherwise likely be euthanized, and (ii) community cat populations (Figure 1). Significant reductions in the intake and euthanasia of cats from targeted areas have been observed at municipal shelters where high-impact targeted TNVR has been implemented (17, 18); shelters employing RTF programs have witnessed sharp, yet comparatively smaller, declines in both measures (19, 20).

In 2012, Best Friends Animal Society received more than $1.6 million in grant funding from PetSmart Charities®, Inc. to begin partnering with municipal shelters across the country to initiate 3-year community cat programs (CCPs), which integrate both RTF and targeted TNVR (Total PetSmart Charities® funding for the six CCPs described in this article was $3.7 million; Best Friends funding was $2.2 million). The CCPs have been generally modeled after the Feral Freedom program, the first large-scale RTF initiative in the U.S., established in 2008 in Jacksonville, Florida, where feline euthanasia was reduced by 92% over 6 years. An important distinction, however, is that the CCPs incorporate both RTF and targeted TNVR components from the onset, whereas in Jacksonville targeted TNVR was not added to RTF efforts until almost 3 years after program inception (20). In the case of the CCPs, targeted TNVR efforts were coordinated (and in large part executed) by Best Friends staff in collaboration with the partner shelters. An examination of one of the inaugural CCPs, in Albuquerque, New Mexico, revealed significant reductions in feline intake and euthanasia over the course of the program, as well as improvements in other associated metrics at the municipal shelter (21). Six CCPs had run to their scheduled conclusions as of year-end 2017. The present study, using various shelter metrics (e.g., feline intake, euthanasia, live-release rate [live outcomes divided by intake (22)], and dead cat collections) summarizes the results of these six CCPs and presents an analysis of the data.

## Materials and Methods

The first two CCPs were initiated at municipal shelters in Albuquerque and San Antonio, Texas, in 2012, followed by the launching of programs at municipal shelters or facilities with municipal sheltering contracts in Baltimore, Maryland, in 2013 and Philadelphia, Pennsylvania, Tucson, Arizona, and Columbus, Georgia in 2014 (Table 1). Programs at each of these open-admission shelters were scheduled to run for 36 months; however, Baltimore, Philadelphia, Tucson, and Columbus were each extended for as many as 3 months because of surplus funds. For the purposes of this investigation, results from only the originally scheduled 3-year program period for each CCP was examined, whether or not the program was extended. In Albuquerque, as described elsewhere (21), a stepwise movement toward the adoption of TNVR as the preferred method of community cat management, including a year-long pilot RTF program at the municipal shelter, preceded the CCP. No formal shelter-based RTF or targeted TNVR initiatives took place prior to the initiation of the CCPs at the other locations.

**Table 1:** Community Cat Program (CCP) locations, shelter name, service areas and size, and program periods.

**CCP location**	**Shelter operator**	**Service area**	Service area size (human population)^*^	**Program period**
Albuquerque, New Mexico	Albuquerque Animal Welfare Department	Bernalillo County	674,000	April, 2012–March, 2015
San Antonio, Texas	San Antonio Animal Care Services	Bexar County	1,826,000	April, 2012–March, 2015
Baltimore, Maryland	Baltimore Animal Rescue and Care Shelter	City of Baltimore	621,000	July, 2013–June, 2016
Philadelphia, Pennsylvania	Animal Care and Control Team of Philadelphia	City of Philadelphia	1,566,000	July, 2014–June, 2017
Tucson, Arizona	Pima County Animal Care Center	Pima County	1,010,000	July, 2014–June, 2017
Columbus, Georgia	Columbus Consolidated Animal Care and Control	Muscogee County	199,000	July, 2014–June, 2017

* *Human population data obtained from U.S. Census Bureau QuickFacts*.

All of the CCPs included integrated implementation of RTF and targeted TNVR components. In general, the RTF component of each CCP was structured so that the vast majority of healthy community cats brought to the shelter from anywhere within their respective service areas, including individuals who could be easily treated for minor injuries or illnesses, were enrolled in the program. Best Friends staff (the number of whom varied by program, but ranged between one and three), arranged for the cats to be sterilized either in-house (when a clinic was present on site) or at a local private high-quality, high-volume spay-neuter clinic. Best Friends personnel, or less frequently, trained volunteers, then returned the cats to the locations where they were trapped. Funding for San Antonio was limited to 14 zip codes; nonetheless, eligible cats brought to the shelter from outside of those zip codes were enrolled into the RTF program and returned to locations of origin by Best Friends staff or volunteers until Program Year 2 when the city began paying for sterilization surgeries and assigning field services staff (often called animal control officers) the task of returning such cats. In Philadelphia, cats were returned to their location of capture by field services staff for the duration of the program.

Before being returned to the field, in addition to being sterilized, all CCP cats were ear-tipped and received vaccinations against rabies and rhinotracheitis/calciviris/panleukopenia (FVRCP), as well as flea treatment and an antibiotic injection (cefovecin sodium, sold under the brand name Convenia®), as appropriate. General protocol called for all free-roaming cats without serious illness or injury to be returned to locations of capture after recovery from sterilization surgery; however, over time, as feline intake declined and more shelter space became available at a number of the CCP locations, some sociable cats were made available for adoption or transferred to private rescue groups (organizations, typically of non-profit tax status, that specialize in the rehoming of adoptable cats). Microchipping was not part of CCP protocol. Relocation (the release of cats at outdoor sites other than location of origin) was not done unless their home environments were deemed too dangerous for safe return (e.g., demolition of a building)—a situation that occurred only rarely.

Targeted TNVR was performed in parts of CCP shelter service areas that were determined to be sources of high feline intake. The methods behind this strategy varied by program inasmuch as each CCP shelter determined how best to allocate and prioritize program resources. For example, Baltimore and San Antonio focused on areas from which the highest frequency or most serious resident complaints were generated, while Philadelphia used admission data to determine locations from which the most cats had been brought to the shelter by residents. Columbus utilized the personal field experience of the program coordinator (who had previously served as the community's animal control officer) to target areas known to be populated by large numbers of community cats until such time that sufficient data was available from the shelter to identify “hot spots” based upon intake numbers alone; targeting hot spots based upon shelter stray cat intake data was also the practice followed by Albuquerque. Tucson concentrated trapping efforts on areas that were identified as sources of high kitten intake. Cats trapped, neutered, and vaccinated as part of targeted trapping efforts were returned to their locations of capture without being admitted to CCP shelters and therefore did not contribute to feline intake totals.

Moreover, in order to make full use of information obtained about the locations of origin of RTF cats, targeted trapping also was performed at RTF release sites when circumstances allowed. Such sites were targeted based upon a hypothesis, known as the “red-flag cat model” which supposes that locations within a community capable of sufficiently supporting one free-roaming cat are likely home to additional unsterilized cats (20, 21). Thus, the initial cat trapped and returned to a new location acts as an indicator, or red flag, alerting program staff to the potential presence of other cats. The red-flag cat model was utilized to varying degrees by all six CCPs. Cats originating from red-flag cat model sites were not separately tracked by the CCPs; however, the number of cats enrolled at each site were tracked by program component (RTF or TNVR) and program year (calendar year for Albuquerque). Therefore, for the purposes of this study, locations at which both RTF and targeted TNVR activity occurred during the same year were categorized as red-flag cat model sites.

Programs of concentrated community outreach were used in the neighborhoods where targeted TNVR took place, including some or all of the following tactics: door-to-door canvassing (a.k.a. block walking), the distribution of door hangers, targeted mass mailings, the hosting of educational events, and the use of cargo vans, wrapped with program-specific messaging, for transport of the cats.

### Data Collection

All CCP-related data were obtained from Best Friends. Procedural details about individual CCPs were obtained via telephone interviews and email correspondence with program coordinators. Dead cat collection data were acquired from individual municipalities or CCP shelters.

CCP staff entered relevant program data (e.g., number of surgeries, sex, age, etc.) into a database built and maintained by Best Friends. Ongoing results were assessed monthly to evaluate the progress of each CCP toward overall sterilization surgery goals. Chameleon software was used to track shelter metrics for Albuquerque, Tucson, and San Antonio; PetPoint software was utilized for Baltimore and Philadelphia; a Lotus Notes program was employed for Columbus. All shelters entered data in real time or on a daily basis.

Shelter metrics tracked specifically as part of the CCPs included live intakes, live outcomes [adoption, transfer to private rescue, return-to-owner (RTO)], and other outcomes (euthanasia, died in care). Intake and euthanasia data were recorded by age: adult and kitten (the age threshold distinguishing kittens from adults varied by CCP, as follows: Albuquerque: ≤ 5 mos.; Baltimore: ≤ 4 mos.; Philadelphia, Tucson, Columbus, and San Antonio: ≤ 6 mos.); admissions of kittens ≤ 2 months of age was tracked separately for Albuquerque, Philadelphia, Tucson, and San Antonio; euthanasia of kittens ≤ 2 months of age was tracked separately for Philadelphia, Tucson, and San Antonio. The number of cats sterilized, whether as part of the RTF or targeted TNVR component of the CCP, as well as the number of cats returned to their trapping sites, adopted, or transferred to private rescue groups were documented. The tracking of welfare outcomes for cats returned to trapping sites was not part of CCP protocol.

### Data Analysis

Shelter cat intake and euthanasia results for 12-month periods matching CCP program dates were compared to a baseline of shelter results for a corresponding 12-month period immediately preceding the initiation of the Albuquerque and San Antonio CCPs, and for the calendar year immediately preceding the Baltimore, Philadelphia, Tucson, and Columbus programs. A similar process was employed to assess results for other shelter metrics (i.e., live-release rate, adoptions, and RTO) as well, except for Albuquerque, for which other metrics were tracked on a calendar-year basis. The number of cats enrolled in the RTF component of each CCP was compared to the number enrolled in the targeted TNVR component for each program year; red-flag cat model results were calculated by matching the number of RTF cats returned to specific sites with the number of cats discovered as a result of targeted TNVR efforts at those same sites and during the same program or calendar year (depending on the available data). Due to the small sample size involved (e.g., 3 program years), varied effort (e.g., returning nearly all RTF cats in the early days of the program while relatively fewer RTF cats were returned later in the program) over the course of the CCP, and inherent year-to-year variation in shelter metrics, no statistical analysis was attempted. Each CCP shelter determined the manner in which to track its data. This was driven largely by the system (e.g., fiscal year, calendar year) used by the municipality itself. The authors acknowledge that uniformity in the tracking of shelter data would have allowed for more straightforward comparisons of some of the results among the various programs.

## Results

### Enrollment and Surgeries

A total of 72,970 cats were enrolled in the six 3-year CCPs. Sterilization surgery was performed on 69,091 (95%) of the enrolled cats. Targeted TNVR conducted as part of the six programs resulted in 54,653 (79%) of the sterilizations, while RTF efforts accounted for 14,439 (21%) of the total surgeries. The combined number of cats sterilized across the six CCPs fluctuated by program year: Year 1: 22,724; Year 2: 24,854; Year 3: 21,513. In aggregate, the percentage of cats sterilized as part of the RTF component of the CCPs decreased each program year: Year 1: 26% (5,881); Year 2: 21% (5,133); Year 3: 16% (3,425) (Tables 2, 3). Overall, the number of female cats sterilized exceeded males 36,184 (52%)−32,907 (48%), and significantly more adults were sterilized than kittens, 49,509 (72%)−19,582 (28%).

**Table 2:** Number of RTF and TNVR surgeries performed annually in each of six 3-year CCPs and percentage of surgery total (in parentheses).

**CCP location (human population)**	**PY1**		**PY2**		**PY3**		**Total surgeries**
	**RTF**	**TNVR**	**RTF**	**TNVR**	**RTF**	**TNVR**	
Albuquerque, NM	964	2,759	759	3,222	464	2,870	11,038
(674,000)	(26)	(74)	(19)	(81)	(14)	(86)	–
San Antonio, TX	877	4,265	238	4,289	245	3,285	13,199
(1,826,000)	(17)	(83)	(5)	(95)	(7)	(93)	–
Baltimore, MD	724	2,803	332	3,299	305	2,804	10,267
(621,000)	(21)	(79)	(9)	(91)	(10)	(90)	–
Philadelphia, PA	1,474	3,299	1,428	2,802	1,152	3,635	13,790
(1,566,000)	(31)	(69)	(34)	(66)	(24)	(76)	–
Tucson, AZ	1,084	2,164	1,642	4,357	736	4,134	14,117
(1,010,000)	(33)	(67)	(27)	(73)	(15)	(85)	–
Columbus, GA	758	1,553	734	1,752	523	1,360	6680
(199,000)	(33)	(67)	(30)	(70)	(28)	(72)	–

**Table 3:** Number of RTF and TNVR surgeries performed annually per 1,000 human residents in each of six 3-year CCPs.

**CCP location (human population)**	**PY1**		**PY2**		**PY3**		**Mean**	
	**RTF**	**TNVR**	**RTF**	**TNVR**	**RTF**	**TNVR**	**RTF**	**TNVR**
Albuquerque, NM	1.4	4.1	1.1	4.8	0.7	4.3	1.1	4.4
(674,000)								
San Antonio, TX	0.5	2.3	0.1	2.3	0.1	1.8	0.2	2.1
(1,826,000)								
Baltimore, MD	1.2	4.5	0.5	5.3	0.5	4.5	0.7	4.8
(621,000)								
Philadelphia, PA	0.9	2.1	0.9	1.8	0.7	2.3	0.8	2.1
(1,566,000)								
Tucson, AZ	1.1	2.1	1.6	4.3	0.7	4.1	1.1	3.5
(1,010,000)								
Columbus, GA	3.8	7.8	3.7	8.8	2.6	6.8	3.4	7.8
(199,000)								

### Disposition

**Figure 2:**
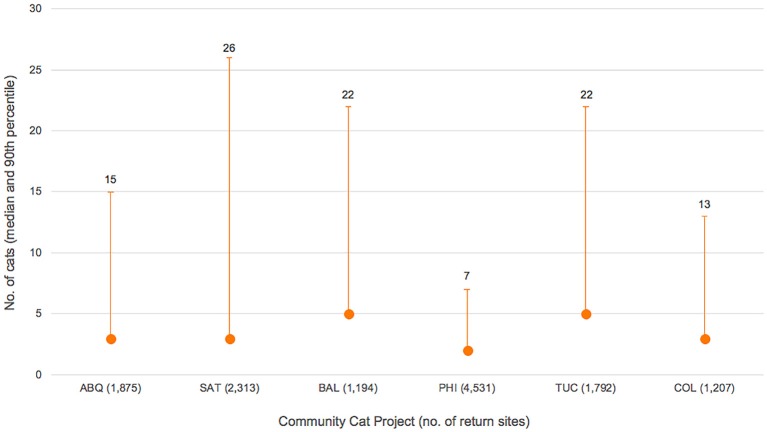
Number of cats corresponding to source/return sites across six CCPs, with median figures (shown as solid dots) giving an approximation of median colony size.

In total, 60,613 cats (83%) were returned to their trapping sites as part of the six CCPs; 10,698 (15%) were adopted or transferred to private rescue; 459 (0.6%) were returned to owner or otherwise released without undergoing surgery; 349 (0.5%) were euthanized for serious health concerns; 204 (0.3%) were relocated because they could not be safely retuned to locations of capture; 140 (0.2%) died perioperatively (Table 4). Of the cats returned to trapping sites, 44,670 (74%) were adults, 13,986 (23%) were kittens and the age of 1957 (3%) was unknown. Cats originated from a total of 12,912 sites across the six programs with the median number of cats per site ranging from 2–5 (Figure 2).

**Table 4:** Disposition of cats in each of the six 3-year CCPs, 3-year totals and percentage by category.

**CCP (human population)**	**RTC (%)**	**Adopt or transfer to rescue (%)**	**RTO (%)**	**Released without surgery (%)**	**Euthanized (%)**	**Relocated (%)**	**Died (%)**	**Other (%)**	Total (%)^*^
Albuquerque, NM	10,738	946	1	1	20	6	34	–	11,746
(674,000)	(91)	(8)	(0.01)	(0.01)	(0.2)	(0.1)	(0.3)	–	(100)
San Antonio, TX	11,904	1,060	0	16	38	75	22	507	13,622
(1,826,000)	(87)	(8)	(0)	(0.1)	(0.3)	(0.6)	(0.2)	(4)	(100)
Baltimore, MD	8,796	2,156	0	11	104	67	24	–	11,158
(621,000)	(79)	(19)	(0)	(0.1)	(0.9)	(0.6)	(0.2)	–	(100)
Philadelphia, PA	12,508	2,085	43	0	93	11	15	–	14,755
(1,566,000)	(85)	(14)	(0.3)	(0)	(0.6)	(0.1)	(0.1)	–	(100)
Tucson, AZ	10,639	3,557	330	4	53	8	32	–	14,623
(1,010,000)	(73)	(24)	(2)	(0.03)	(0.4)	(0.1)	(0.2)	–	(100)
Columbus, GA	6,028	894	22	31	41	37	13	–	7066
(199,000)	(85)	(13)	(0.3)	(0.4)	(0.6)	(0.5)	(0.2)	–	(100)
Total	60,613	10,698	396	63	349	204	140	507	72,970
	(83)	(15)	(0.5)	(0.1)	(0.5)	(0.3)	(0.2)	(0.7)	(100)

* *Some totals exceed 100% due to rounding; RTC, returned to colony; cats released without surgery had already been sterilized; Other, unspecified outcome*.

### Euthanasia and Intake

**Figure 3:**
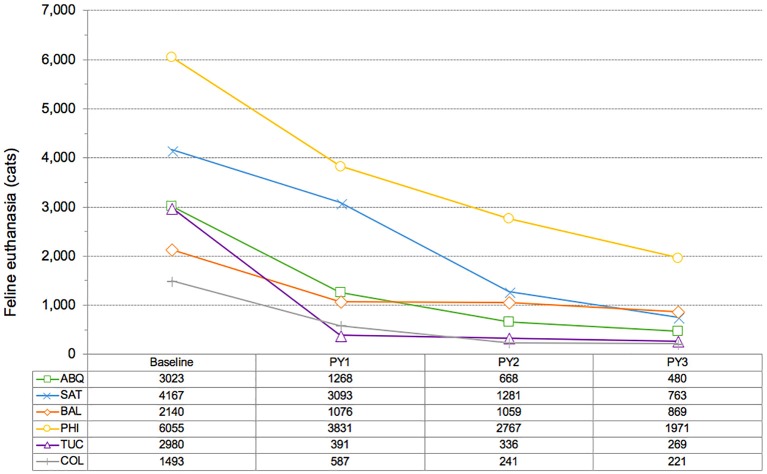
Changes in feline euthanasia at six CCP shelters, comparison of baseline level to euthanasia during each of three program years (PY).

A median decline of 83% in overall feline euthanasia occurred at the six shelters when results from the end of the third year of each program are compared to baseline results (Table 5 and Figure 3). Tucson observed the largest decline in euthanasia on a percentage basis (91%) while Philadelphia experienced the largest drop in absolute numbers (4,084 cats). Among the six CCPs, Baltimore experienced the smallest percentage decrease in the euthanasia of cats (59%); Columbus had the smallest decline in absolute terms (1,272 cats). Over the same periods, the euthanasia of kittens declined by a median of 87%; the euthanasia of “newborn” kittens (≤ 2 months) fell by a median of 85% at the three shelters (Philadelphia, San Antonio, and Tucson) where such data were tracked. The largest decline in the euthanasia of kittens, both on a percentage basis and in absolute terms, was observed by Tucson (95% and 2,305 cats, respectively), while the smallest reduction, by either measure, occurred at Baltimore (64% and 364 cats, respectively). Euthanasia of cats per 1,000 residents in each of the respective shelter's service areas declined by a median of 84%; on the same basis, kitten euthanasia declined by a median of 87% (Table 6).

**Table 5:** Common shelter metrics before and after implementation of each 3-year CCP (absolute numbers and percentages by category).

**Shelter metrics**	**CCP location**											
	**Albuquerque**		**San Antonio**		**Baltimore**		**Philadelphia**		**Tucson**		**Columbus**	
	**Before**	**After (% change)**	**Before**	**After (% change)**	**Before**	**After (% change)**	**Before**	**After (% change)**	**Before**	**After (% change)**	**Before**	**After (% change)**
Feline intake	9,776	6,102 (−38)	6,661	6,581 (−1)	6,977	5,999 (−14)	19,017	12,791 (−33)	7,635	5,266 (−31)	3,329	1,842 (−45)
Per 1,000 residents	15	9 (−40)	4	4 (0)	11	10 (−9)	12	8 (−33)	8	5 (−38)	16	9 (−44)
Kittens^*^^†^	4,441	2,468 (−44)	3,810	4,283 (12)	2,978	1,823 (−39)	8,868	5,313 (−40)	5,072	2,903 (−43)	1,487	1,104 (−26)
≤ 2 mos. of age^†^	2,803	1,672 – (40)	2,706	4,241 (57)	–	–	5,729	3,347 (−42)	4,479	2,143 (−52)	–	–
Feline euthanasia	3,023	480 (−84)	4,167	763 (−82)	2,140	869 (−59)	6,055	1,971 (−67)	2,980	269 (−91)	1,493	221 (−85)
Per 1,000 residents	5	1 (−80)	2	0.4 (−80)	3	1 (−67)	4	1 (−75)	3	0.3 (−90)	7	1 (−86)
Kittens^*^^†^	1,462	149 (−90)	2,489	340 (−86)	568	204 (−64)	2,372	493 (−79)	2,424	119 (−95)	669	84 (−87)
≤ 2 mos. of age^†^	–	–	1,875	276 (−85)	–	–	1,965	360 (−82)	2,327	113 (−95)	–	–
Euthanasia rate (%)	31	8 (−74)	63	12 (−81)	32	15 (−53)	32	15 (−53)	39	5 (−87)	45	12 (−73)
Kittens^*^^†^ (%)	33	6 (−82)	65	8 (−88)	19	11 (−42)	27	9 (−67)	48	4 (−92)	45	8 (−82)
Live release rate (%)	61	90 (48)	31	83 (168)	63	79 (25)	63	74 (17)	51	83 (63)	54	85 (57)
Adoptions	4,264	3,333 (−22)	893	1,947 (118)	3,228	2,648 (−18)	4,853	4,911 (1)	3,375	3,682 (9)	380	68 (−82)
RTO	297	277 (−7)	69	139 (101)	54	84 (56)	150	228 (52)	140	111 (−21)	43	45 (5)
DOA cats	2,220	1,689 (−24)	8,002	10,299 (29)	4,215^‡^	3,336^‡^ (−21)	712^§^	328^§^ (−54)	575	495 (−14)	N/A	N/A –

* *Kitten definitions varied by shelter: Albuquerque ≤ 5 mos*.

*Baltimore ≤ 4 mos.; San Antonio, Philadelphia, Tucson, Columbus ≤ 6 mos*.

† *All kittens in Albuquerque, regardless of age, tracked by calendar year (year-end 2011 to year-end 2015), rather than program year*.

‡ *Cat and dog data combined (no further breakdown available)*.

§ *Only DOA cats brought to the shelter by the public are included; data for those picked up by municipality were unavailable*.

**Table 6:** Impact of CCPs on shelter feline intake and euthanasia per 1,000 human residents.

**Common shelter metrics**	**CCP location**					
	**Albuquerque**	**San Antonio**	**Baltimore**	**Philadelphia**	**Tucson**	**Columbus**
Mean annual sterilizations per 1,000 human residents	5	2	6	3	5	11
**FELINE INTAKE Per 1,000 HUMAN RESIDENTS**						
Before program	15	4	11	12	8	16
After program	9	4	10	8	5	9
Change (%)	−40	0	−9	−33	−38	−44
**FELINE EUTHANASIA Per 1,000 HUMAN RESIDENTS**						
Before program	5	2	4	4	3	7
After program	0.7	0.4	1	1	0.3	1
Change (%)	−86	−80	−75	−75	−90	−86
KITTEN^*^ **INTAKE Per 1,000 HUMAN RESIDENTS**						
Before program	7	2	5	6	5	7
After program	4	2	3	3	3	6
Change (%)	−43	0	−40	−50	−40	−14
KITTEN^*^ **EUTHANASIA Per 1,000 HUMAN RESIDENTS**						
Before program	2	2	0.9	2	2	3
After program	0.2	0.2	0.3	0.3	0.1	0.4
Change (%)	−90	−90	−67	−85	−95	−87
**DEAD CATS COLLECTED Per 1,000 HUMAN RESIDENTS**						
Before program	3.3	4.4	6.8^^†^^	0.4	0.6	N/A
After program	2.7	5.9	5.8^^†^^	0.2	0.5	N/A
Change (%)	−17	34	−15^^†^^	−50	−17	N/A

* *Kitten definitions varied by shelter: Albuquerque ≤ 5 mos.; Baltimore ≤ 4 mos.; San Antonio, Philadelphia, Tucson, Columbus ≤ 6 mos. Kitten data was tracked by program year for all CCPs, except Albuquerque, where it was tracked only by calendar year*.

† *Reflects collection of all dead animals—no break down by species available. Before program = 12-month period immediately preceding program period for Albuquerque and San Antonio (except for Albuquerque kitten data); calendar year immediately preceding year of program initiation for Baltimore, Philadelphia, Tucson, and Columbus*.

**Figure 4:**
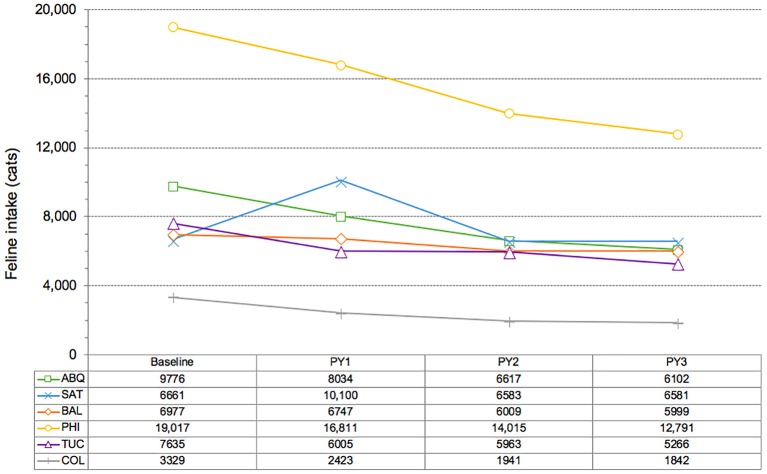
Changes in feline intake at six CCP shelters, comparison of baseline level to intake during each of three program years (PY).

Overall feline intake dropped by a median of 32% at the six shelters; Columbus experienced the largest decline (45%) while the smallest decline (1%) in feline intake was observed at San Antonio (Table 5 and Figure 4). Kitten intake declined by a median of 40% across the six shelters, while the admission of newborn kittens dropped by a median of 41%, at the four facilities (Albuquerque, Philadelphia, San Antonio, and Tucson) for which such data were available. Overall feline intake fell by a median of 33% per 1,000 residents across the six CCPs, while a 40% drop in the intake of kittens occurred (Table 6).

### Live-Release Rate

The live-release rate for cats at the six CCP shelters increased by a median of 53% over the 3-years of the CCPs. The largest gain, 168%, was at San Antonio (from 31 to 83%). Philadelphia observed the smallest increase (17%, from 63 to 74%); however, the baseline live-release rate there was, by comparison, more than double that of San Antonio (Table 5).

### Adoptions

Changes in the absolute number of cats adopted over the course of the six CCPs varied significantly (median of −8%), ranging from an increase of 118% for San Antonio to a decline of 82% for Columbus (Table 5). Measured as a proportion of feline intake, however, the adoption rate for cats increased (median of 45%) at all locations (in large part due to reductions in feline intake), except for Baltimore (−5%). When the number of cats transferred to private rescue groups for adoption are added to the adoptions originating directly from the shelters themselves, increases (median of 39%) were observed at all CCP locations.

### RTO

In aggregate, the number of RTO cats increased by 17%, from 753 to 884 cats across the six CCPs, although Albuquerque (297–277) and Tucson (140–111) experienced declines. Median RTO as a percentage of shelter feline intake increased from 1.2% prior to CCP inception to 2% after completion of the respective programs.

### Red-Flag Cat Model

A total of 15,658 cats (22% of the total cats enrolled in the six CCPs) originated from 1,817 red-flag cat model sites, where both RTF and targeted TNVR took place during the same 12-month period. Almost two thirds of these were TNVR cats (10,297), which amounts to 19% of all cats sterilized as part of targeted TNVR efforts. On average, 4 TNVR cats (median of 2) were enrolled in CCPs for each RTF cat returned to red-flag cat model locations.

### DOA

Data for cats classified as “dead on arrival” (DOA) were mixed across the six CCPs, and comparisons were made difficult due to uneven tracking and reporting (Table 5). Albuquerque and Tucson, for example, documented reductions of 24 and 14%, respectively. Baltimore observed a 21% reduction in the *total* number of dead animals picked up, but no breakdown by species was available. The most significant reduction (54%) was associated with Philadelphia; however, the only data available were for “stray” cats brought to the shelter by the public as DOA; no data for cats picked up by the municipality were available. As a result, the total number of DOA cats remains unknown for this CCP. San Antonio, by contrast, observed a significant increase (29%) in DOA cats over the course of the CCP. A year-by-year breakdown, however, shows an initial increase of 36% from 2011 to 2012 followed by a 17% decrease from 2012 to 2015, roughly mirroring the initial increase in feline intake and subsequent decline (Figure 4). No data were available for Columbus.

## Discussion

### Impact of CCPs on Feline Euthanasia and Intake

As has been documented in other communities where RTF programs have been implemented at open-admission municipal shelters (19–21), significant reductions in feline euthanasia (median of 83%) were observed across all six CCPs (Figure 3). The declines in overall feline euthanasia at four of the six CCP shelters (Albuquerque, Tucson, San Antonio, and Columbus) exceeded 80% over 3 years, surpassing reductions witnessed over 4-year periods in Jacksonville and San José, where RTF programs resulted in reductions of approximately 70% (19, 20, 23). Even larger declines in the euthanasia of kittens (median of 87%) occurred at all CCP locations. Despite significant differences in the communities served by the six CCP shelters, both in terms of geography and population size, each experienced sharp declines in feline euthanasia, which strongly corroborates previous research (19, 21). Integration of targeted TNVR with RTF appears to be generally associated with more rapid declines in euthanasia. Results after 32 months (including an 8-month pilot period) of an ongoing CCP in Las Vegas, Nevada, further support these findings (as with the other CCPs, data for the Las Vegas program was obtained from Best Friends), as feline euthanasia dropped by 80% (from 8,439 to 1,705) at the facility there, which provides municipal animal care and control services. RTF surgeries (5,748) represent 66% of total Las Vegas program sterilization surgeries (8,704 or 4 per 1,000 residents) over this period.

In addition, the feline euthanasia rate (calculated by dividing the number of cats euthanized for reasons other than owner request by the total number of live feline intakes) dropped by a median of 74% across CCP locations. A median euthanasia rate of 36% existed before integrated RTF and targeted TNVR programs began; the same measure at the conclusion of the respective CCPs was 12%. As a point of reference, Shelter Animals Count reported for 2016 a feline euthanasia rate of 25% (calculated by dividing the total number of cats euthanized, less owner-requested euthanasia, by the total number of outcomes minus owner-requested euthanasia) among its 627 participating organizations categorized as municipal shelters or organizations with municipal sheltering contracts. Shelter Animals Count functions as a national database of sheltered animals and follows the Base Data Matrix specified by the National Federation of Humane Societies; all data are contributed on a voluntary basis and were self-reported by 3,535 total participant organizations, which included municipal shelters and shelters with government contracts, as well as rescue groups with government contracts and shelters and rescues without such contracts, in 2016 (24).

Reductions in feline intake (median of 32%) across the six CCP shelters (Figure 4) varied more than reductions in euthanasia. As stated above, the largest reduction occurred at Columbus (45%), while San Antonio experienced the smallest decline (1%) over the course of the 3-year program. A spike of 52% in feline intake during Year 1 at San Antonio was followed by a reduction in Year 2 (35%) that approximated the median decline (33%) experienced at the other CCP locations over the entirety of their programs; intake was virtually flat in Year 3 of the San Antonio program, declining by just 2 cats. Possible explanations for the anomalous increase in feline intake experienced during San Antonio's first year include a particularly sharp increase in awareness of community cats among the residents there and, a surge in the use of the municipal shelter as a resource for cats, due at least in part to new perceptions among residents of the shelter as a “cat-friendly” facility (20). Additional factors that might have contributed include the faster movement of cats in and out of the facility as cats returned to the field typically spent no more than 24 h in care at the shelter, rather than being kept for 4 days (prior to likely euthanasia) as was the practice before initiation of the CCP. Unfortunately, a definitive explanation for the increase in intake during the first program year of the San Antonio CCP was not readily apparent from the available evidence.

Notwithstanding the initial spike in intake witnessed by San Antonio, the median decline in overall feline intake among the six CCPs surpassed in 3 years the reductions in intake observed over 4-year periods in Jacksonville and San José (similar to the results for euthanasia noted above), where such declines were 30 and 27%, respectively. Again, implementation from the onset of concurrent RTF and targeted TNVR programs is the likely reason for these favorable results. The ongoing CCP in Las Vegas provides additional evidence in support of the strong association between such integrated community cat management programs and rapid reductions in feline intake: the Las Vegas shelter observed a 39% decline (from 13,424 to 8,220) in feline intake 32 months after the implementation of CCP protocols.

The median reduction in the intake of kittens (40%) at the six CCP shelters exceeded the median drop in total feline intake (32%), with Albuquerque observing the largest decline (44%). San Antonio was the only program to see an overall increase in kitten intake (12%), which occurred in a fashion similar to what was previously described concerning total feline intake, whereby a surge in the admission of kittens (69%) happened in year one, followed by a combined decline of 33% during years 2 and 3 of the program. Significant reductions in feline intake associated with targeted TNVR efforts have been documented elsewhere and attributed to “several factors” (17). However, the dramatic reductions in kitten intake in particular, documented across all six CCPs, suggests an impact (the extent of which is, admittedly, unknown) on reproductive capacity in the CCP service areas, since any other programs that might account for the observed reductions (e.g., diverting kittens to private rescue groups without admission to the shelter) were implemented only on a small scale where they existed at all.

### Impact of CCPs on Other Shelter Metrics

As stated above, live-release rate increased significantly (median of 53%) across all six CCPs. The median live-release rate at the six shelters increased from 57% prior to CCP inception to 83% after the completion of the respective programs; post-CCP live-release rates (range: 74–90%; Table 5) compare favorably to a live-release rate of 69% for municipal shelters and shelters with government contracts participating in the Shelter Animals Count database in 2016 (24).

Post-CCP RTO rates (2%) were below the average RTO rate for municipal shelters and shelters with government contracts participating in the Shelter Animals Count database in 2016 (3%) (25), but consistent with results from a national survey of U.S. households, which found that 2% of lost cats were recovered by contacting a local shelter (26). Multiple survey-based studies have indicated that the most common method by which lost cats are reunited with their owners is cats returning home on their own (26, 27). Consequently, it is likely that an unknown percentage of cats returned as part of RTF efforts were actually lost pets who, at some point after being returned, found their way back to their owners (and likely at a rate of reunification greater than would have occurred had these cats been admitted to the shelter).

### Impact of CCPs Compared to Similar Programs in Other Communities

The size of the human population served by each of the six CCP shelters varied, from ~200,000 (Columbus) (28) to almost 1.9 million (San Antonio) (29), and fluctuations of up to 8% in population size took place over program periods at some sites (29). To account for these differences in population size, feline intake (Table 7) and euthanasia (Table 8) results were also examined on a normalized (per 1,000 human residents) basis. Median reductions in feline intake (33%) and euthanasia (84%) calculated in this manner varied little from median reductions (32 and 83%, respectively) derived from the absolute intake and euthanasia data reported above. A comparison of these results with those from Jacksonville and San José (Tables 7, 8) found that the median 3-year decline in intake at CCP shelters exceeded reductions over the same number of years in Jacksonville (30%) and San José (26%). The median reduction in euthanasia per 1,000 human residents at CCP sites also surpassed declines over the same period in both Jacksonville (71%) and San José (69%). Unlike the CCPs, which featured fully integrated RTF and targeted TNVR elements throughout, RTF was the primary focus of the programs in Jacksonville and San José; however, a formalized targeted TNVR component (as noted above) was added to the Jacksonville program in its third year, and an *ad hoc* targeting effort similar to the red-flag cat model utilized at CCP sites was operated concurrently with the RTF initiative in San José. The specific impact of targeted TNVR efforts on results produced by the RTF-based programs in Jacksonville and San José is difficult to quantify; however, based upon the greater median reductions in intake and euthanasia at CCP locations, the benefits of combining targeted TNVR and RTF are apparent. Results of a 2-year targeted TNVR campaign in Alachua County, Florida offer the clearest evidence of the impact of targeting on feline intake and euthanasia at a municipal shelter. A 69% reduction in intake and a 95% decline in euthanasia occurred in the targeted area (zip code 32601) vs. reductions of 13% in intake and 30% in euthanasia for the remainder of the county, where no targeting took place (17) (Tables 7, 8). The totality of these results suggests that the integration of targeted TNVR and RTF programs exhibits the greatest capacity for reducing the intake and euthanasia of cats on a community-wide scale.

**Table 7:** Annual reduction in feline intake for each of six 3-year CCPs per 1,000 human residents in each corresponding shelter service area, and comparison to similar programs in other communities.

**Community/program (source)**	**Baseline**	**Year 1**	**Year 2**	**Year 3**	**Year 4**	**Year 5**
Albuquerque	15	12	10	9	–	–
San Antonio	4	6	4	4	–	–
Baltimore	11	11	10	10	–	–
Philadelphia	12	11	9	8	–	–
Tucson	8	6	6	5	–	–
Columbus	16	12	10	9	–	–
San José (17)	10	9	8	7	8	7
Jacksonville (21)	16	15	15	11	11	11
Alachua, target (15)	13	9	4	–	–	–
Alachua, non-target (15)	16	15	14	–	–	–

*Baseline = 12-month period immediately preceding program period for Albuquerque and San Antonio; calendar year immediately preceding year of program initiation for all others*.

**Table 8:** Annual reduction in feline euthanasia for each of six 3-year CCPs per 1,000 human residents in each corresponding shelter service area, and comparison to similar programs in other communities.

**Community/Program (source)**	**Baseline**	**Year 1**	**Year 2**	**Year 3**	**Year 3**	**Year 5**
Albuquerque	5	2	1	0.7	–	–
San Antonio	2	2	0.7	0.4	–	–
Baltimore	3	2	2	1	–	–
Philadelphia	4	3	2	1	–	–
Tucson	3	0.4	0.3	0.3	–	–
Columbus	7	3	1	1	–	–
San José (17)	7	6	3	2	2	2
Jacksonville (21)	13	11	7	4	4	3
Alachua, target (15)	8	2	0.4	–	–	–
Alachua, non-target (15)	10	7	7	–	–	–

*Baseline = 12-month period immediately preceding program period for Albuquerque and San Antonio; calendar year immediately preceding year of program initiation for all others*.

### Analysis of Source/Return Site Characteristics

Cats originated from a total of 12,912 unique sites across the six CCPs, with medians for individual CCPs ranging from 2 to 5 cats (Figure 2). These values are less than those documented by Nutter in rural North Carolina (median: 10 cats across 11 discrete colonies) (13), Natoli et al. in Rome, Italy (median: 12 cats across 103 discrete colonies) (30), and Tan et al. in urban parts of Australia (median: 12 cats across 44 discrete colonies (31), but comparable to those documented in an urban Chicago, Illinois, neighborhood (median: 0–6 cats across 20 discrete colonies) (12). Data from the present study are not necessarily inconsistent since the median values from the previous studies refer to colony size prior to sterilization efforts and were based upon colony censuses. The CCP data, by contrast, reflect only the number of cats enrolled in the CCPs.

Examination of source/return site data reveals that the maximum number of cats returned to a single location can be deceiving. Data from Albuquerque, for example, show that 205 cats originated from one site: a mobile home community (approximately 0.33 km^2^ in size) for which shelter staff used a common address when recording intake (and, as appropriate, return) information. Similar situations were observed in other CCP communities. For this reason, 90th percentile (as opposed to maximum) was chosen to represent the upper-end of the number of cats present at each source/return site. Results of this analysis correspond well with those of Natoli et al. who reported that colonies of 21 or more cats were uncommon in Rome, Italy (30).

### Implications of the Red-Flag Cat Model

As stated above, on average, 4 TNVR cats (median of 2) were enrolled in CCPs for each RTF cat returned to red-flag cat model locations; these results are similar to what was previously documented by Albuquerque (where such information was tracked by calendar year) (21). It was not uncommon for a dozen or more cats to be enrolled at the same location as a result of targeted TNVR in response to a single cat being brought to the shelter; one site targeted by San Antonio had 116 cats enrolled in such a fashion, which is illustrative of the potential of the red-flag cat model (and integration of RTF and targeted TNVR programs in general). The red-flag cat model was employed as part of each CCP as staffing and circumstances on the ground allowed, which varied by program location; for example, Baltimore enrolled the most TNVR cats across the greatest number of red-flag cat model sites during Year 1, while Columbus experienced this peak in Year 2 and Philadelphia and Albuquerque in Year 3 (Tucson and San Antonio saw the number of red-flag cat model sites and total number of TNVR cats trapped at such sites peak in different program years).

### General Health of Cats Enrolled in the CCPs

Consistent with what has been observed at other locations where RTF (19) and targeted TNVR (17) programs have been implemented, the cats enrolled in all of the CCPs were generally in good health, as was evidenced by the low incidence of cats requiring euthanasia due to serious health concerns (0.5%) or dying in care (0.2%). As mentioned above, the welfare outcomes for cats returned to locations of origin were not tracked as part of the CCPs; in fact, little research on this topic could be found. A single example was uncovered from a published report describing the RTF program in Jacksonville, where for more than a year at the beginning of the program cats were microchipped for the purpose of tracking the number that “would be hit by cars… starve to death, be attacked by dogs, and many other hypothetical tragedies that should nullify the program” (32). The report concluded: “After more than a year of such identification absolutely none of the more than 6,000 feral cats with a microchip were ever identified as falling into any of those theoretical situations” (32). Indeed, the microchipping of cats as part of the Jacksonville RTF program was discontinued when “no evidence of mistreatment of returned cats turned up” (20). Further research in to the welfare outcomes associated with cats of shelter origin returned to the field after sterilization and vaccination is warranted. Considerable data, however, including what has been reported above, have been published in support of the assertion that community cats are in generally good health upon enrollment in programs that revolve around TNVR and its variants (12, 17, 19, 21, 33).

### Analysis of DOA Data

DOA data from Albuquerque and Tucson (reductions of 24 and 14%, respectively) were comparable to the 20% reduction (from 1,629 to 1,308) reported following 4 years of RTF in San José (19) (Table 5). San Antonio documented many more DOA cats than any other CCP (more than 20 times that of Tucson). Neither the initial increase (described previously) nor the greater overall DOA numbers could be explained by those who provided the data. The reductions observed by Albuquerque and Tucson—as well as those suggested by the “combined” data from Baltimore and incomplete data from Philadelphia—would seem to support the hypothesis that targeted sterilization efforts decreased the number of community cats in CCP service areas, and is consistent with evidence from elsewhere suggesting that neutered male cats “lose interest in mating with females which considerably reduces their inclination to roam” (19, 34–36). The data from San Antonio, however, are less consistent. Given the increasing popularity of TNVR (37) and RTF programs (25) and concerns for the welfare of cats being returned (38), this is an important area of investigation for future studies.

## Limitations

As has been encountered elsewhere (12, 21, 39), the limitations of the present study include those commonly experienced when conducting a retrospective investigation, which is bound by the constraints of the available data. For instance, some types of data were tracked differently across the CCPs: overall feline intake, euthanasia, euthanasia rates, and surgery counts were tracked by program year for all six locations, but Albuquerque tracked other metrics (e.g., live-release rate, RTO, kitten results) only by calendar year; baseline results for Albuquerque and San Antonio reflect 12-month periods immediately preceding program initiation, whereas baselines presented for Baltimore, Philadelphia, Tucson, and Columbus reflect end-of-year results for the calendar year immediately preceding those programs. Cats originating from red-flag cat model sites were not separately tracked by the CCPs; however, the number of cats enrolled at each site were tracked by program component (RTF or TNVR) and program year (calendar year for Albuquerque). Therefore, for the purposes of this study, locations at which both RTF and targeted TNVR activity occurred during the same year were categorized as red-flag cat model sites. Moreover, shelter metrics were not formally tracked by zip code; therefore, an assessment of the impact of targeted TNR on intake and euthanasia for specific zip codes, as has been formulated elsewhere (17), was not attempted.

Community cats were enrolled in the CCPs as they were discovered and trapped or brought into the shelters. Return site information, including location and the surgery records of individual cats, was entered into an internal Best Friends database. Such information was updated throughout the program as cats were trapped, sterilized, and returned; however, records of the number of cats at each colony site upon entry into the CCP are incomplete. Therefore, assessment of changes in colony size over the course of the program was not possible. In addition, the welfare outcomes for cats returned to sites of origination were not specifically recorded, precluding analysis.

## Conclusions

Significant and rapid reductions of feline euthanasia and intake occurred across all CCPs (the single anomaly being the largely unexplained rise in intake during Year 1 of the San Antonio program), highlighting the effectiveness of integrating RTF and targeted TNVR. Use of the red-flag cat model, which was employed as part of all CCPs, improved the efficiency of targeted TNVR efforts. It was found that cats enrolled via the RTF and targeted TNVR components of all CCPs were in good general health, corroborating prior research (17, 21, 33). In general, the number of cats found at source/return sites was small, which is consistent with results of previous research conducted on community cats residing in urban environments (12, 30). Although cat-specific DOA data were not obtainable for all locations, the available evidence generally supports the hypothesis that significant declines in dead cat collections suggest a combination of fewer community cats and reduced roaming on the part of sterilized individuals (19).

## Data Availability

The raw data supporting the conclusions of this manuscript will be made available by the authors, without undue reservation, to any qualified researcher.

## Author Contributions

PW conceived of the research idea. DS collected and examined the data. Both authors wrote/edited the paper.

### Conflict of Interest Statement

In recognition of Frontiers policy and our ethical obligation as researchers, the authors acknowledge that the funding sponsors provided general guidance for the design of the study and were periodically apprised of project status during data collection, analysis, and interpretation; and the writing of the manuscript. We further acknowledge that one of the authors (PW) is employed by Best Friends Animal Society, advocating for the protection of domestic cats via public policy initiatives, and the other (DS) works under a contractual arrangement with Best Friends and Michelson Found Animals Foundation, Inc.
